# Birth rate after major trauma in fertile-aged women: a nationwide population-based cohort study in Finland

**DOI:** 10.1186/s12978-022-01387-w

**Published:** 2022-03-24

**Authors:** Matias Vaajala, Ilari Kuitunen, Lauri Nyrhi, Ville Ponkilainen, Maiju Kekki, Tuomas T. Huttunen, Ville M. Mattila

**Affiliations:** 1 Faculty of Medicine and Life Sciences, University of Tampere, Tampere, Finland; 2 Department of Pediatrics, Mikkeli Central Hospital, Mikkeli, Finland; 3 Institute of Clinical Medicine and Department of Pediatrics, University of Eastern Finland, Kuopio, Finland; 4 Department of Surgery, Central Finland Central Hospital Nova, Jyvaskyla, Finland; 5 Department of Obstetrics and Gynecology, Tampere University Hospital Tampere, Tampere, Finland; 6 Center for Child, Adolescent and Maternal Health Research, Faculty of Medicine and Health Technology, Tampere University, Tampere, Finland; 7 Department of Anesthesia and Intensive Care, Tampere University Hospital and Tampere Heart Hospital, Tampere, Finland; 8 Department of Orthopaedics and Traumatology, Tampere University Hospital, Tampere, Finland

## Abstract

**Background:**

To date, only a few small studies have assessed the effects of major orthopedic traumas on the subsequent birth rate in fertile-aged woman. We assessed the incidences of traumatic brain injury (TBI) and fractures of the spine, pelvis, and hip or thigh and evaluated their association with the birth rate in fertile-aged woman.

**Methods:**

In this retrospective register-based nationwide cohort study, data on all fertile-aged (15–44 years of age) women who sustained a TBI or fracture of the spine, pelvis, hip or thigh between 1998 and 2013 were retrieved from the Care Register for Health Care. A total of 22,780 women were included in TBI group, 3627 in spine fracture group, 1820 in pelvic fracture group, and 1769 in hip or thigh fracture group. The data were subsequently combined with data from the National Medical Birth Register. We used Cox regression model to analyze the hazard for a woman to give birth during 5-year follow-up starting from a major trauma. Women with wrist fractures (4957 women) formed a reference group. Results are reported as hazard ratios (HR) with 95% confidence intervals (CI).

**Results:**

During 5-year follow-up after major trauma, 4324 (19.0%) women in the TBI group, 652 (18.0%) in the spine fracture group, 301 (16.5%) in the pelvic fracture group, 220 (12.4%) in the hip or thigh fracture group, and 925 (18.7%) in the wrist fracture group gave birth. The cumulative birth rate was lower in the hip or thigh fracture group in women aged 15–24 years (HR 0.72, CI 0.58–0.88) and 15–34 years (HR 0.65, CI 0.52–0.82). Women with pelvic fracture aged 25–34 years also had a lower cumulative birth rate (HR 0.79, CI 0.64–0.97). For spine fractures and TBIs, no reduction in cumulative birth rate was observed. Vaginal delivery was the primary mode of delivery in each trauma group. However, women with pelvic fractures had higher rate of cesarean section (23.9%), when compared to other trauma groups.

**Conclusions:**

Our results suggest that women with thigh, hip, or pelvic fractures had a lower birth rate in 5-year follow-up. Information gained from this study will be important in clinical decision making when women with previous major trauma are considering becoming pregnant and giving birth.

**Supplementary Information:**

The online version contains supplementary material available at 10.1186/s12978-022-01387-w.

## Plain language summary

To date, only a few small studies have assessed the effects of major orthopedic traumas on the subsequent birth rate in fertile-aged woman. We assessed the incidences of traumatic brain injury (TBI) and fractures of the spine, pelvis, and hip or thigh and evaluated their association with the birth rate in fertile-aged woman.

Data on all fertile-aged (15–44 years of age) women who sustained a TBI or fracture of the spine, pelvis, hip or thigh between 1998 and 2013 were retrieved from the Care Register for Health Care and the data was then subsequently combined with data from the National Medical Birth Register.

A total of 22,780 women were included in TBI group, 3627 in spine fracture group, 1820 in pelvic fracture group, 1769 in hip or thigh fracture group, and 4957 in wrist fracture group, which was used as control group. Of these, 4324 (19.0%) women in the TBI group, 652 (18.0%) in the spine fracture group, 301 (16.5%) in the pelvic fracture group, 220 (12.4%) in the hip or thigh fracture group, and 925 (18.7%) in the wrist fracture group gave birth during the 5-year follow-up.

Our results suggest that women with thigh, hip, or pelvic fractures had a lower birth rate in 5-year follow-up. Information gained from this study will be important in clinical decision making when women with previous major trauma are considering becoming pregnant and giving birth.

**Supplementary Information:**

The online version contains supplementary material available at 10.1186/s12978-022-01387-w.

## Introduction

Traumas to the head, spine, pelvis, and femur are usually caused by high-energy impact, such as vehicle collisions and falls from height [1–4]. In particular, traumatic brain injuries (TBI) are one of the most common and socially notable traumas [5]. Moreover, the mortality rates of people suffering especially severe TBIs are higher compared to the general population [6, 7]. In the younger population, however, the incidence of spine, pelvic and hip trauma is not as high as that of head trauma [8–10]. The mortality rate following hip and pelvic trauma is known to be relatively low in the younger population, ranging between 1.3% and 3.5% among the population aged 18–49 years [10].

In Finland, there has been an increasing trend in the incidence of TBI, spine, and pelvic trauma [8]. Indeed, the average incidence of hospitalized TBI for all women during the years 1991–2005 was 80 per 100,000 person-years, an increase of 59% [7]. The incidence of spine fractures leading to hospitalization in all patients over 20 years of age in Finland increased from 57 per 100,000 person-years in 1998 to 89 per 100,000 person-years in 2017 [8]. Moreover, among Finnish adults, the incidence of pelvic fractures increased from 34 to 56 per 100,000 person-years between 1997 and 2014 [11].

Although the incidences and effects of major trauma on health have been studied extensively, there is a scarcity of studies on the effects of major trauma on fertility among women. Many earlier studies have focused mainly on trauma and abnormalities of the reproductive system, especially of the uterus and ovaries [12]. It has been reported, however, that musculoskeletal trauma around the area of the pelvic ring and the femur can cause sexual dysfunction and dyspareunia [13, 14]. Moreover, women in Finland who have undergone total hip replacement are reported to have a lower birth rate than women in the general population [15].

Our hypothesis is that major trauma can affect sexuality and sexual function and thereby increase the threshold for becoming pregnant and reduce the number of births. The aim of this nationwide register study is therefore to report the incidence of TBIs and fractures of the spine, pelvis, and hip or thigh in fertile-aged women in Finland and to investigate the effects of these injuries on the birth rate.

## Materials and methods

In this retrospective nationwide register-based cohort study, data were obtained from the Care Register for Health Care, which has a coverage of more than 95% [16], and the National Medical Birth Register (MBR), which has a coverage of nearly 100% [17, 18]. The study period was from 1998 to 2018.

Data on deliveries and newborns after major orthopedic trauma were collected from the MBR, which contains information on all pregnancies, delivery statistics, and the perinatal outcomes of births with a birthweight of ≥ 500 g or a gestational age of ≥ 22^+0^. Our data included all pregnancies and deliveries from fertile-aged (15–49 years of age) women during our study period. The variables used in this study are defined in the MBR register description [19].

All fertile-aged (15–49 years of age) women with TBI, spine fracture, pelvic fracture, or hip or thigh fracture occurring during the study period were identified from the Care Register for Health care. We used women who were hospitalized with fracture of the wrist as a reference group. Women with fractures of the wrist were chosen as a reference group because we expected these women to be similar in background and risk-taking behavior to those women in the major trauma groups than women in the general population without any injuries. In addition, as wrist fractures generally heal quickly, we did not expect them to have a major impact on fertility, and therefore they formed a good reference group.

**Fig. 1:**
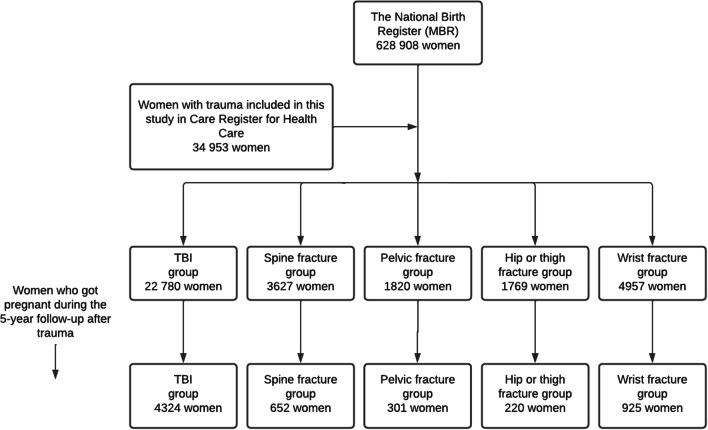
Flowchart of the study populations for Cox regression analysis. Data from the MBR were combined with data on the diagnosed major traumas in the Care Register for Health Care

ICD-10 (International Classification of Diseases 10th revision) codes were used to identify the trauma patients. The specific ICD-10 codes with definitions for each major trauma group and reference group included in this study are shown in Additional file 1: Table S1. Due to challenges in distinguishing new traumas and control visits/appointments, the first trauma hospitalization for a woman in each category was included (meaning that the same woman can be included in multiple study groups). The formation of the study groups and number of women who became pregnant during the 5-year follow-up after the first trauma is described in Fig. 1. In the evaluation of pregnancy outcomes after different traumas, each pregnancy found in our data after traumas (1998–2018) was included.

Due to the best possible comparability between major trauma groups, the annual incidences during our study period were calculated using the same criteria, despite the varied nature of the different traumas included in the study. Therefore, for each trauma group, only the first hospitalization period with trauma diagnosis per patient was classified as a separate trauma, as the control appointments could occur after a long period, making it unreliable to identify subsequent traumas in the Care Register for Healthcare.

The base population used for the calculation of the birth rate and incidences of major traumas was the number of females aged 15–49 who were living in Finland at the end of a particular year. The population data were obtained from Statistic Finland. During our study period, the size of the study population decreased from 1,389,409 in 1998 to 1,285,100 in 2018. The annual number of newborns was also obtained from Statistic Finland (stat.fi) [20].

### Ethics

Both the National Medical Birth Register (MBR) and the Care Register for Health Care used the same unique pseudonymized identification number for each patient. The pseudonymization was performed by the Finnish data authority Findata. The authors did not have access to the pseudonymization key as it is maintained by Findata. In accordance with Finnish legislation, no informed written consent was required because of the retrospective register-based study design and because the patients were not contacted. Permission to use this data was granted by Findata after evaluation of the study protocol (Permission number: THL/1756/14.02.00/2020).

### Statistics

Continuous variables were reported as mean with standard deviation or as median with interquartile range based on distribution of the data. Categorized variables were presented as absolute numbers and percentages. The annual birth rate was calculated using the size of the base population of fertile-aged (15–49 years) women living in Finland at the end of a particular year (31.12) and the number of yearly newborns. The base population for the incidences of different traumas were all women aged 15 to 49 who were living in Finland at the end of a particular year. Base population figures were obtained from Statistic Finland (stat.fi) [20]. The Cox regression model was used to evaluate the risk for the first live-born child in women after major trauma in relation to reference individuals with wrist fracture. The results were interpreted with hazard ratios (HRs) and 95% confidence intervals. Proportional hazards assumption was tested using Schoenfeld residuals and the supposition was true. To control the confounding effect of age, women with trauma were divided into three categories based on their age at the time of trauma: the categories were 15–24, 25–34, and 35–44 years. The start of the follow-up was the date of the trauma in the Care Register for Health care. The endpoint of the follow-up was the first live-born child after the trauma, or the common closing date, which was 5 years after the trauma. Because a 5-year follow-up period is required for the Cox regression model, all women with a trauma occurring after 2013 were excluded from the survival analysis because the follow-up period after this is not fully available based on the data. Moreover, as 49 is the maximum age for fertile-aged woman in this study, the required 5-year follow-up condition of fertile years is only met by women who sustained trauma before the age of 45. Statistical analysis was performed using R version 4.0.3.

## Results

**Fig. 2:**
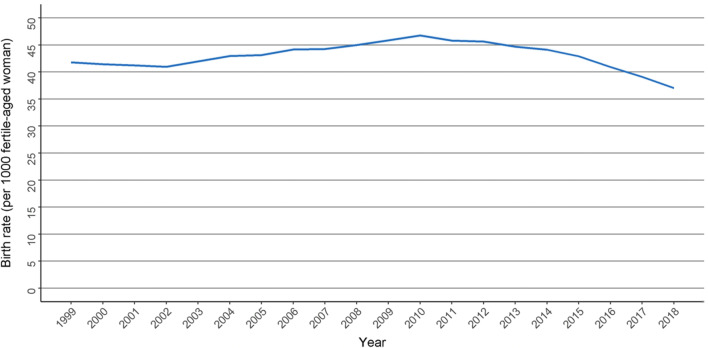
Birth rate with 95% confidence intervals per 1000 for the whole Finnish population of fertile-aged (15–49 years) women during the study period

Initially, the annual birth rate for the whole population of fertile-aged women showed an increasing trend during our study period, rising from 41.1 newborns per 1000 fertile-aged woman in 1998 to 46.8 per 1000 fertile-aged women in 2010, but then decreased strongly to 37.0 per 1000 fertile-aged women in 2018. The average annual birth rate between 1998 and 2018 was 42.9 (Fig. 2).

**Fig. 3:**
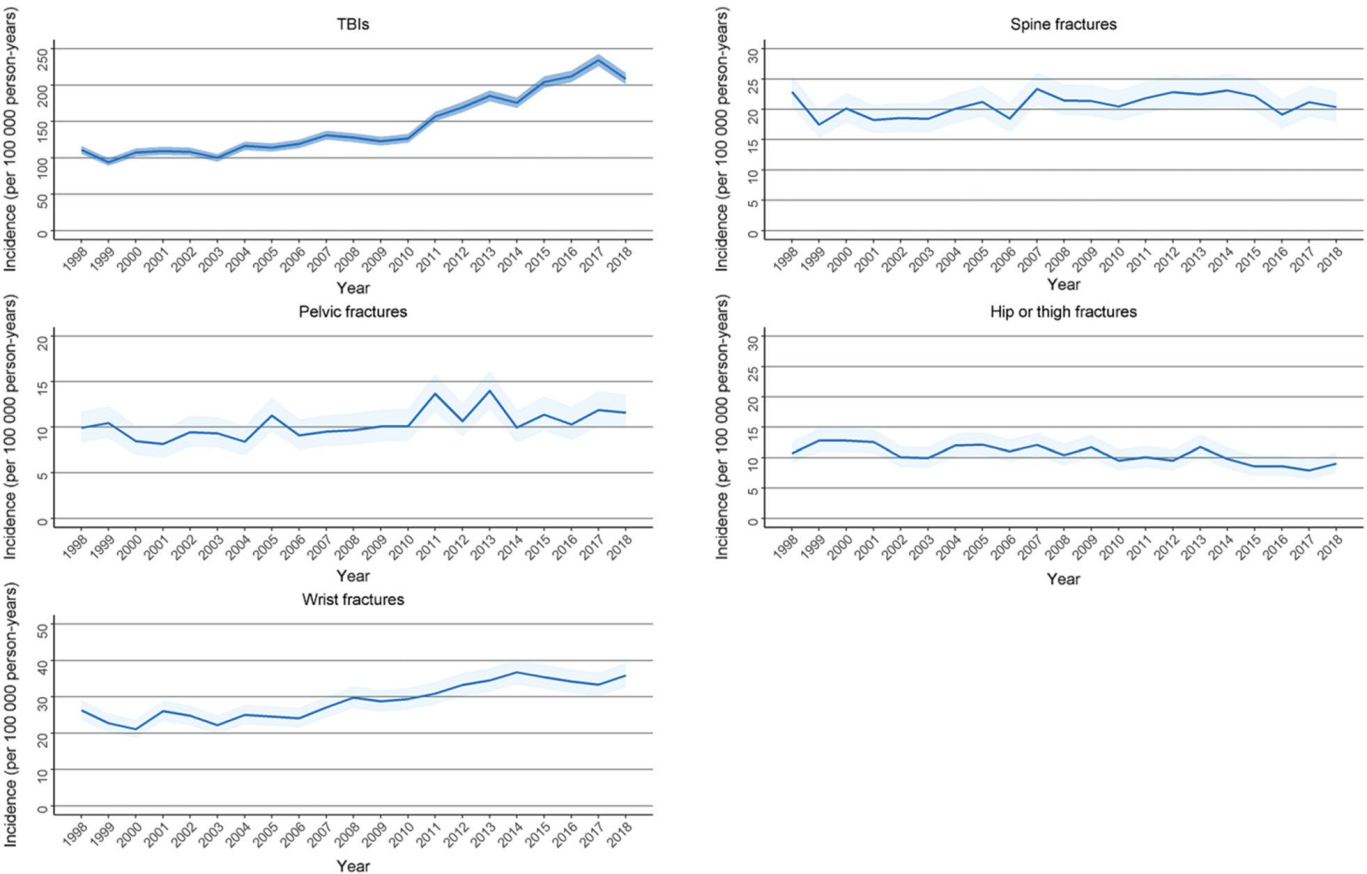
Incidence with 95% confidence intervals of major traumas and the reference group (wrist fractures) in women (15–49 years) included in this study

During the study period, the incidence of TBIs, which originally also had a notably higher incidence than the other traumas included in this study, showed a strongly increasing trend, increasing from 110.9 per 100,000 person-years in 1998 to 208.8 per 100,000 person-years in 2018. Furthermore, the incidence of wrist fractures increased from 26.3 per 100,000 person-years in 1998 to 35.9 per 100,000 person-years in 2018. The incidence of hip or thigh fractures, pelvic fractures, and spine fractures remained stable during our study period, ranging between 7.9 and 12.8 per 100,000 person-years for hip or thigh fractures, 8.1 and 14.0 per 100,000 person-years for pelvic fractures, and 17.5 and 23.4 per 100,000 person-years for spine fractures (Fig. 3).

Women in the hip or thigh fracture group had the lowest birth rate during the 5-year follow-up period after fracture (12.4%). The highest birth rate during the 5-year follow-up was in the TBI group (19.0%), which was also higher than in the reference group (18.7%) (Table 1). Women in the hip or thigh fracture group had lower hazard for the event of giving birth during the 5-year follow-up period in the 15–24 years (HR 0.72, CI 0.58–0.88) and the 25–34 years (HR 0.65, CI 0.52–0.82) age groups when compared to the wrist fracture group. Furthermore, women in the pelvic fracture group aged 25–34 had lower hazard for giving birth during the 5-year follow-up period (HR 0.79, CI 0.64–0.97). Spine fractures and TBIs did not show an impaired cumulative birth rate when compared to wrist fractures (Table 2).

**Table 1:** Background information on the study groups and the reference group (wrist fractures) for the survival analysis

	TBI group	Spine fracture group	Pelvic fracture group	Hip or thigh fracture group	Wrist fracture group
Total number of women included*	22,780	3627	1820	1769	4957
Age at the start of follow-up					
15–24 years	10 273 (45.1%)	1476 (40.7%)	852 (46.8%)	707 (40.0%)	2004 (40.4%)
25–34 years	5965 (26.2%)	1018 (28.1%)	481 (26.4%)	437 (24.7%)	1430 (28.8%)
35–44 years	6542 (28.7%)	1133 (31.2%)	487 (26.8%)	625 (35.3%)	1523 (30.7%)
Number of women giving birth during the 5-year follow-up (%)	4324 (19.0%)	652 (18.0%)	301 (16.5%)	220 (12.4%)	925 (18.7%)
Age at the time of trauma (mean; SD)	27.6 (9.2)	28.5 (9.1)	27.4 (9.2)	28.4 (9.1)	28.4 (9.1)
Age at the time of delivery (mean; SD)	28.0 (5.6)	28.5 (5.5)	27.9 (5.4)	28.7 (5.4)	28.7 (5.4)
Follow-up period in weeks (mean; SD)	237.6 (55.6)	237.8 (56.3)	240.9 (52.3)	246.4 (44.6)	237.9 (55.6)

*Because a 5-year follow-up period was required, only women with trauma occurring before 2014 and aged under 45 years at the time of trauma were included for Cox survival analysis

**Table 2:** Age-stratified hazard ratios (HR) with 95% confidence intervals (CI) for women giving birth in the major trauma groups of this study

Age	15–24 years	25–34 years	35–44 years
TBI group			
Hazard ratio (CI)	1.09 (0.98–1.21)	0.92 (0.83–1.02)	0.99 (0.76–1.29)
Spine fracture group			
Hazard ratio (CI)	1.02 (0.88–1.17)	0.91 (0.78–1.06)	1.06 (0.74–1.51)
Pelvic fracture group			
Hazard ratio (CI)	0.91 (0.77–1.09)	0.79 (0.64–0.97)	0.67 (0.39–1.18)
Hip or thigh fracture group			
Hazard ratio (CI)	0.72 (0.58–0.88)	0.65 (0.52–0.82)	0.60 (0.35–1.01)

The major trauma groups were compared with all fertile-aged women with wrist fractures during the same study period

When compared to other trauma groups, the rate of cesarean sections after fractures was highest in the pelvic fracture group (23.9%), followed by TBI group (20.3%), hip or thigh fracture group (20.3%) and spine fracture group 20.2%. The wrist fracture group had the lowest rate of cesarean section (18.2%). However, despite the preceding trauma, vaginal delivery was the primary mode of delivery in all trauma groups. There was a relatively high proportion of fetuses in all trauma groups who were exposed to maternal smoking during pregnancy compared to the average rate for the whole Finnish population (23.5–27.1% vs 14.6%). Previous CS rate was similar between groups (9.7–11.7%).

## Discussion

The main finding of this study was that younger women with hip or thigh fractures had (evidently) a lower hazard of giving birth during the follow-up period. In addition, there was a considerable variation in the rates of women giving birth during the follow-up period, when compared to the wrist fracture group. The cumulative birth rate was a little lower for women aged 25–34 with pelvic fracture. When compared with women with wrist fractures, spine fractures or TBIs did not have a substantial effect on the birth rate during the 5-year follow-up after major trauma. During our study period, the incidence of TBI hospitalizations in Finland increased strongly among fertile-aged women. This study is unique in that it gives baseline information on the effects of major traumas on the subsequent birth rate.

When compared to wrist fractures, hip or thigh fractures and pelvic fractures were the only major traumas included in this study that had a negative impact on the birth rate during the five subsequent years after sustaining the fracture. There are a few studies that have reported sexual dysfunction in women with proximal thigh traumas or pelvic traumas, with sexual dysfunction occurring mostly among younger women [13, 14]. However, a study on proximal thigh traumas reported that in most cases only a few women report anything other than mild or no sexual dysfunction after 1-year follow-up [14]. In addition, dyspareunia is commonly reported, especially after fractures of the pelvic ring [21]. These factors could most likely explain the lower hazard in these two groups. However, based on our data, the exact reason remains unknown. As the number of women in the hip or thigh and pelvic fracture groups was lower than in the other groups in this study, this might have influenced the results.

One likely explanation is the fear of possible negative outcomes resulting from previous trauma of the pelvic area or femur which may result in women choosing not to get pregnant or deciding not to give birth vaginally. Based on our results, however, spontaneous vaginal delivery was the primary mode of delivery after traumas, as only 18–24% of the deliveries after trauma in each trauma group were cesarean sections. However, the rate of cesarean sections in trauma groups was little higher when compared to general rate in Finland (16–17%) [19]. The findings of this study should serve to reduce any doubts mothers may have of their capability to go through pregnancy and give birth after major trauma. As for other TBIs and spine fractures, the hazard for giving birth was the same as that of wrist fractures, which suggests that these traumas do not have a negative effect on fertility or subsequent pregnancies. Moreover, we are unaware of previous studies that report sexual dysfunction caused by spine fractures or TBIs.

The incidence of TBI hospitalization increased strongly among fertile-aged women. This finding can be mostly explained by indirect temporal factors and phenomena, such as the significant increase in the amount of CT imaging (Stuk.fi [22]) and an improved awareness of mild TBIs (especially concussions [23]), which lowers the patient-based threshold to seek medical care [24]. Furthermore, the creation of a joint emergency service in 2011 may have also led to improvements in acute head trauma diagnostics.

The strength of our study is the large nationwide study population with a long study period, which made it possible to compare large patient groups. The register data used in our study are routinely collected with structured forms with national instructions, which ensures good coverage and reduces possible reporting and selection bias [25]. Furthermore, the coverage of both registers included in this study is high [16]. To our best knowledge, this study is the first to examine the effects of a variety of major traumas on the subsequent capability of women to become pregnant and give birth using large national research material with uniform delivery-related guidelines and attitudes.

The main limitation of our study is the missing clinical information on the TBIs and fractures included in this study (e.g., radiological finding). As this information is not recorded to the registers, we could only use ICD-10 coding, which means that the severity of the traumas remains unknown. Further, our ICD-10 codes were limited to trauma-related codes, meaning that other factors possibly affecting the outcome during or before the follow-up period also remain unknown. Due to these limiting factors, the effects of trauma severity or possible polytraumas on birth-rate remains unknown.

## Conclusion

Our results suggest that giving birth was more challenging for women with thigh, hip, or pelvic fractures in 5-year follow-up. However, neither TBIs nor spine fractures negatively affected the possibility of having a child during 5-year follow-up. Information gained from this study should be considered by women and physicians when a woman who has sustained major trauma is considering the possibility and possible risks of becoming pregnant and giving birth.

## Supplementary Information

**Additional file 1: Supplementary Table 1:** ICD-10 codes with definitions for each major trauma group and reference group included in this study.

## Data Availability

Data used in this study cannot be shared without the permission of the Finnish authority Findata.

## Abbreviations

CS: Cesarean section
TBI: Traumatic brain injury
MBR: Medical Birth Register
HR: Hazard ratio
CI: Confidence intervals
