# Supplementary material for: Birth rate after major trauma in fertile-aged women: a nationwide population-based cohort study in Finland
Source: Reprod Health. 2022 Mar 24;19:73. doi: 10.1186/s12978-022-01387-w (PMC8944167; doi:10.1186/s12978-022-01387-w)
Supplement: Supplementary file 1 — Additional file 1: Supplementary Table 1: ICD-10 codes with definitions for each major trauma group and reference group included in this study. [file 12978_2022_1387_MOESM1_ESM.docx]

Supplementary Table 1: ICD-10 codes with definitions for each major trauma group and reference group included in this study.

| TBI |  |
| --- | --- |
| ICD-10 code | Definition |
| S06.0 | Concussion |
| S06.1 | Traumatic cerebral edema |
| S06.2 | Diffuse traumatic brain injury |
| S06.3 | Focal traumatic brain injury |
| S06.4 | Epidural hemorrhage |
| S06.5 | Traumatic subdural hemorrhage |
| S06.6 | Traumatic subarachnoid hemorrhage |
| S06.8 | Other specified intracranial injuries |
| S06.9 | Unspecified intracranial injury |
| Spine fractures |  |
| ICD-10 code | Definition |
| S12.0 | Fracture of first cervical vertebra |
| S12.1 | Fracture of second cervical vertebra |
| S12.2 | Fracture of third cervical vertebra |
| S12.7 | Multiple fractures of cervical vertebra |
| S12.8 | Fracture of other parts of neck |
| S12.9 | Fracture of neck, unspecified |
| S22.0 | Fracture of thoracic vertebra |
| S22.1 | Multiple fractures of thoracic vertebra |
| S32.0 | Fracture of lumbar vertebra |
| Pelvic fractures |  |
| ICD-10 code | Definition |
| S32.1 | Fracture of sacrum |
| S32.3 | Fracture of ilium |
| S32.4 | Fracture of acetabulum |
| S32.5 | Fracture of pubis |
| S32.7 | Multiple fractures of lumbar spine and pelvis |
| S32.8 | Fracture of other parts of pelvis |
| S32.9 | Fracture of unspecified parts of lumbosacral spine and pelvis |
| Hip or thigh fractures |  |
| ICD-10 code | Definition |
| S72.0 | Fracture of head and neck of femur |
| S72.1 | Pertrochanteric fracture |
| S72.3 | Fracture of shaft of femur |
| S72.4 | Fracture of lower end of femur |
| S72.7 | Multiple fractures of femur |
| S72.8 | Other fracture of femur |
| S72.9 | Unspecified fracture of femur |
| Wrist fractures |  |
| ICD-10 code | Definition |
| **S62.0** | Fracture of navicular bone of wrist |
| S62.1 | Fracture of other and unspecified carpal bone |
| S62.2 | Fracture of first metacarpal bone |
| S62.3 | Fracture of other and unspecified metacarpal bone |
| S62.4 | Multiple fractures of metacarpi |
